# Game-based learning in medical education

**DOI:** 10.3389/fpubh.2023.1113682

**Published:** 2023-03-03

**Authors:** Maosen Xu, Yong Luo, Yu Zhang, Ruolan Xia, Hong Qian, Xiuhe Zou

**Affiliations:** ^1^Division of Biotherapy, Cancer Center, West China Hospital, Sichuan University, Chengdu, China; ^2^Department of Head and Neck Oncology, West China Hospital, Sichuan University, Chengdu, China; ^3^Department of Cardiovascular Surgery, West China Hospital, Sichuan University, Chengdu, China; ^4^Department of Thyroid Surgery, West China Hospital, Sichuan University, Chengdu, China

**Keywords:** game-based learning, serious game, gamification, medical education, teaching activity

## Abstract

At present, medical education is rapidly evolving. Game-based learning (GBL) has been gradually used for education, and several innovations have emerged. The emergence of serious games and gamification provides alternative approaches for educators to improve the medical teaching process. Both serious games and gamification exert their education-promoting function by providing the possibility of combining learning activities such as feedback, testing, and spaced repetition with active participation and autonomy as well as positive experiences for students. Developing effective GBL modalities has the potential to bring immersive experiences for medical students and improve their study outcomes. Herein, we reviewed recent studies employing GBL in medical education, including serious games and gamification teaching. Furthermore, we also discussed the effectiveness and limitations of GBL to suggest future directions for the development and application of GBL in medical education.

## 1. Introduction

With the rapid development of modern technologies and the iterations of educational concepts, the repertoire of untraditional strategies for education is becoming wider gradually. Among these novel and appealing methods, game-based learning (GBL) is fast developing as an interesting and innovative teaching approach in the field of education currently (1). Over the recent years, mounting evidence suggests that GBL can improve engagement and stimulate the motivation of students to learn, as well as promote teaching outcomes (2–8).

At present, GBL is a new trend and is extensively applied in a wide spectrum of disciplines. Given the actuality that medical students are more receptive to new things and keen on modern technologies, various interesting games are also more frequently used in medical teaching (9, 10). As opposed to more traditional instructional approaches, the main modality of GBL is adding diverse game elements to other non-game areas to encourage engagement and raise the enthusiasm of participants. The scope of GBL is extensive, and it covers both non-technological and technological integration of games within the teaching activity (1). GBL is typically referred to as “serious games,” “educational games,” and “gamification” in both undergraduate courses and clinical studies. The formal definition of serious games is an interactive computer application that has a challenging goal and incorporates some scoring mechanism, therefore providing users with practical knowledge, skills, or attitudes in real life (11, 12). Gamification is another strategy of GBL, and it can be described as a process of game-thinking and game mechanics to engage users and solve problems (13, 14). Both these methods are currently used to promote medical education.

Simulation-based education (SBE) is another teaching strategy that can provide a relatively safe practice environment for both learners and patients in the field of medical education. SBE has the potential to help learners acquire technical skills and non-technical skills including leadership, teamwork ability, and communication (15–17). Despite having similar purposes and application prospects, the differences between SBE and GBL remain. The main model of GBL is adding game elements to other non-game areas to encourage engagement, which emphasizes the application of game elements in teaching activity and finally improves teaching results (1). However, medical simulation is broadly categorized into four areas, namely, a partial-task simulator, a screen-based computer, standardized patients, and high-fidelity mannequin simulators. By using these teaching tools, SBE largely allows learners to practice their clinical skills without patients being put at risk (15).

It is now becoming increasingly recognized that the application of game elements in education will provide an engaging and enjoyable way of learning and contribute to valuable improvements in studying outcomes. Furthermore, the appearance and epidemic of COVID-19 exerts a profound influence on medical education and enables educators to search for viable pedagogical models continuously (18). To avoid a large-scale epidemic of COVID-19 and provide appropriate social distancing, numerous educative institutions had to undergo shutdown to varying degrees. In this condition, GBL can help students to enrich their study experience and enhance their collaboration during social distancing. Therefore, the application of gamified elements in education has enormous potential to create an educational and engaging studying journey for students and can be seen as a promising option to enrich current teaching methods.

In this review, we overview recent studies employing GBL in medical education, including serious games and gamification teaching. Furthermore, we also discuss the effectiveness and limitations of GBL, to suggest future directions for the development and application of GBL in medical education.

## 2. Methods

We performed a PubMed search using the following search terms: “game-based learning AND medical education,” “serious game AND medical,” “educational games AND medical,” and “gamification AND medical.” We reviewed and assessed all abstracts of those searched literature carefully to select appropriate articles including original articles, meta-analyses, and reviews. Meanwhile, we excluded articles that do not belong to the field of medical education. As a result, we made summaries according to different categories in the next sections.

## 3. Serious games in medical education

Apart from entertainment function, another purpose of serious games is to be a pedagogical tool, which provides a way of interactive learning for medical education and constitutes a balanced combination between learning activity and amusement (11). Serious games include playing elements to support educational objectives deliberately and are generally illustrated as digital games that inform, educate, and train (19). It can also be done in different formats such as digital, card, and board games (20). The benefits of serious games on learners include enhancing their collaborative awareness *via* multiplayer settings, providing them with opportunities for active learning to better solve clinical problems, and improving their clinical reasoning, decision-making skills, clinical performance, and the like (5, 21, 22). As such, serious games have potential alternatives for supplementing traditional simulation-based education and contributing to better academic performances of medical students (23). Various serious games in medical teaching activity together with their main proposes and advantages are listed in Table 1.

**Table 1:** Serious games in medical teaching activity.

**Game name**	**Filed**	**Game type**	**Country and year**	**Game propose**	**Advantages**	**Reference**
Cleft Island	Surgery	Online game	Thailand, 2019	To develop and evaluate the effectiveness of serious game to deliver knowledge	Improving students knowledge	(24)
EMERGE	Surgery	Online game	Germany, 2019	To test the effect of the game on students' declarative and procedural knowledge, as well as their satisfaction.	Increasing students declarative knowledge	(25)
PediatricSim	Pediatry	Online game	America, 2018	To teach and assess students performances on several critical pediatric scenarios	Improving students engagement and test result	(26)
NEOGAMES	Pediatry	Online game	China, 2020	To train students in neonatal resuscitation and to examine whether the game improves their long-term knowledge retention	Facilitating learning and promoting short-term and long-term knowledge retention	(27)
AntibioGame^®^	Lemology	Online game	France, 2019	To evaluate the usability and playability of the game	Improving students knowledge in antibiotic prescription	(28)
Hygie	Continuing medical education	Prototype video game	France, 2019	To evaluate the effectiveness and satisfaction of the game	Promoting CME in an effective, pleasant, and evidence-based way.	(29)
“spaced-education” game	Continuing medical education	Electronic questionnaire	America, 2012	To assess the efficacy of the game as a method of CME among physicians.	Substantially improving guidlines knowledge and is a well-accepted method by students	(30)
InsuOnline©	Continuing medical education	Questionnaire	Brasil, 2015	To improve the knowledge of undergraduate students and medicine residents about insulin therapy	A good option for large-scale continuing medical education on diabetes.	(31)
Fydlyty	Quality-oriented education	Online game	Canada, 2017	To improve medical-based cultural competence education	Providing an engaging, easily accessible and modifiable, and cost-effective cultural competence training tool	(32)
Happy families	Physiotherapy education	Card game	Belgium, 2022	To assist learners to develop their clinical reasoning proficiency and help learners develop adaptive expertise	Improving students clinical reasoning capacity	(33)
GridlockED	Emergency	Board game	Canada, 2019	To identify teaching points to which learners are exposed while playing the game.	Creating opportunities for engaging medical learners in systems-level teaching	(34)
COVIDgame	Epidemiology	Offline Game	China, 2021	To explore the effectiveness of the game for improving medical students' COVID-19 knowledge	Improving students knowledge retention	(35)
eMedOffice	Medical practice	Computer-aided game	Germany, 2011	To teach medical students the organizational and conceptual basics of the medical practice of a general practitioner	Promoting the future development of more effective serious games	(36)
APS Game	Health care education	Online game	Brazil, 2019	To compare the influence of the game dedicated to primary health care with traditional learning methods on students knowledge	Improving the students' knowledge effectively	(37)

The primary objectives of research regarding serious games in medical education are assessing their playability, practicability, and pedagogical outcomes for some specific contexts and diseases, and widespread efforts have been put into this. In the field of surgical medical teaching, some enjoyable games have been designed and obtained good results. For example, the game “Cleft Island” was developed to test whether learners could benefit from and gain related medical knowledge from this studying format as well as measure the game experience of students (24). The game development is made up of three components including “mechanic,” “dynamic,” and “aesthetic,” which aims to give participants a better experience. The results demonstrate that this game can be used as an effective supplementary instructional material and improve students' knowledge of treatment protocol (24). Another game, named “EMERGE,” was applied to explore the implication of GBL on students' declarative and procedural knowledge, which was achieved by the design of a pretest-posttest manner (25). Specifically, students were asked to complete questions concerning declarative and procedural knowledge before and after playing the “EMERGE,” respectively. The result demonstrated that this pretest-posttest comparison increased students' knowledge and yielded a positive impression of them. However, this research failed to test the long-term effects of this game (25).

Serious games are also used in the course of pediatric teaching. Recently, Gerard et al. designed a first-person serious game termed “PediatricSim” that was intended to teach and evaluate students' performances in different pediatric settings such as respiratory failure, diabetic ketoacidosis, and septic shock (26). By selecting from distant treatment options, all participants were put in the role of a code leader and direct patient management to improve their cognizance of pediatric emergencies. Furthermore, this survey also assesses the engagement of students and the educational value of “PediatricSim.” It is gratified that most subjects rated the game favorably for engagement, educational value, and realism (26). Another game “NEOGAMES” aimed at promoting pediatric education to improve long-term knowledge retention of students and successfully train students in neonatal resuscitation in a cost-friendly and accessible manner (27).

Limited curricular time typically hinders teaching effects of infectious diseases. As a complementary device, serious games contribute to efficaciously pedagogical outcomes by creating highly immersive experiences for users. A serious game, AntibioGame^®^, integrates various game techniques and elements including cartoon graphics, mascots, and avatars, which aim to improve the training of medical students in the antibiotic application and test its usability and playability. As a result, 96% of students liked it and all students said they would recommend it to others (28).

In addition to imparting expertise, serious games also play crucial roles in medical-based cultural competence education. By providing an engaging, easily accessible, and modifiable training tool, the game “Fydlyty” is shown to be intuitive and promote medical-based cultural competence education. It incorporates a scenario editor and dialogue authoring tool as well as employs low-fidelity visuals, which enables educators and students to access it directly in a more intuitive and simple way (32). Furthermore, serious games can also be used to teach medical residents for improving their management and leadership skills, since many of them think that leadership and negotiation are two additional domains they need (38).

There are multiple serious game modalities in the type of experiences created for medical students. Different from online games that consisted of complicated elements, both card games and board games have the potential to generate ideal teaching outcomes in a low-cost and more available manner. Hage et al. designed a comprehensive and structured card game “Happy Families” that enhanced learning enjoyment and promoted the cognition of students by making learners analyze subjective and physical examinations of a given patient (33). GridlockED is a multiplayer cooperative board game designed to assist learners in understanding the concepts of prioritization and patient flow in the emergency department. The investigators also tried to identify teaching points when students are playing the GridlockED game (34). These innovative teaching formats are emerging as complementary devices and represent promising options for classes. As for their long-term efficiency and applicability in veritably clinical practices, further investigations are needed.

The epidemic of COVID-19 has reshaped public awareness and posed great challenges for the field of medical education. One of the implications on education is that COVID-19 leads to large-scale disruption of university teaching activity (39). In this context, it is necessary to develop innovative approaches for medical teaching about basic knowledge of COVID-19 because most medical students will face and treat patients with COVID-19. To enrich teaching activities and help students to acquire COVID-19 knowledge effectively, Hu et al. designed a novel game named “COVIDgame” and compared its effectiveness in improving medical students' COVID-19 knowledge with online lectures (35). The game is composed of three separate parts including choosing the right order for putting on and taking off personal protective equipment, recognizing patients with COVID-19 promptly, and assessing confirmed patients. Only when every step is right can players accomplish the game. The final test scores demonstrate that the use of serious games improves students' study outcomes and contributes to improving knowledge retention (35).

Continuing medical education (CME) is an important part of medical education, especially for general practitioners. However, limited learning strategies together with their time-consuming disadvantage usually impede the further development of CME. In some cases, the use of technology creates more realistic situations by providing subjects with immersive experiences and finally achieving improved scores. Therefore, CME games have the potential to make up traditional methods tedious and sporadic. At present, the objectives of most CME games are to verify their effectiveness and satisfaction (29–31). By comparing the game with article reading, a traditional continuing education, researchers demonstrated that serious games functioned in a more effective, pleasant, and evidence-based way (29). Even though the outcome of serious games sometimes is not encouraging as hoped, it is at least as effective as a traditional educational activity and is proposed to be a feasible option for large-scale CME (31).

The advantages of using serious games in education not only include enriching pedagogical formats, promoting students' engagement, and enhancing their learning outcomes effectively as mentioned earlier, but also have the potential to reduce expensive bedside teaching and optimize the design of medical practices (36, 40, 41). Despite these benefits, challenges and limitations remain. The application of serious games fails to generate ideal results in regard to transferring learners' clinical reasoning skills acquired in serious games to other cases addressing similar clinical problems (42). Furthermore, the feasibility and effects of serious games require a match between task complexity and the learner's competency level in some cases because a game may not be effective for novices but be useful for experts, which is also known as the “expertise reversal effect” (43). Therefore, measures should be taken to ensure that junior trainees learn when playing the game. Cost and efficiency are important factors that need to be considered while developing a feasible game for teaching activity since excellent software created by third-party designers even costs thousands of dollars. In addition to the economic expenditure, the time needed for training instructors as well as the need for technical support and resources are common barriers that need to be solved (1). In addition, some research designs concerning serious games in teaching activity are not following the principles of randomization and control, which makes it difficult to exclude other factors influence. Notwithstanding, we can see that some randomized controlled trials are conducted continually. These trials confirmed the positive implications of serious games on teaching knowledge (37, 44).

## 4. Gamification in medical education

Gamification is another strategy of game-based learning, which can be described as the application of the characteristics and benefits of games to real-world problems and a process of game-thinking and game mechanics to engage users and solve problems (13, 14). The field of gamification in medical teaching activity is innovative and developed constantly. Even though gamification and serious game have some analogous characteristics and conceptual overlap, there are some means to distinguish them. The easiest and most direct method is removing the game elements to see whether the learning activity still functions. Also, different from the combination of learning and game goals in serious games, gamification is a design technique that typically layers game goals on top of learning goals to motivate engagement and constructive behavior of participants (45).

It has been recognized that the use of gamification in medical education is significant and salutary. Meanwhile, the area of related study is expanding. Several published articles comprehensively summarized its advantages such as enhancing collaboration and increasing the engagement of students, improving their earning analytic and clinical decision-making capacity, as well as offering them opportunities for deliberate practice in clinical reasoning (13, 46). In light of its profound influences, gamification has been widely applied in various medical disciplines and different learning stages, particularly for the millennial learner, to promote significant teaching outcomes and to further evaluate its effectiveness, which is listed in Table 2.

**Table 2:** Gamification in medical teaching activity.

**Game name**	**Filed**	**Game type**	**Country and year**	**Propose**	**Advantages**	**Reference**
NO	Microbiology	Online question bank	America, 2022	To promote students meaningfully engagement and increase their knowledge base	Improving students class exam scores and engagement.	(47)
NO	Microbiology	Checkerboard game	India, 2021	To assess the perception of students regarding game in enhancing learning process.	Fostering learning process and cognition of medical students in the microbiology course.	(48)
SIDRA	Anatomy of locomotor system	Online game	Spain, 2020	To engage students and improve their educational performance	Better student responses and academic performance.	(49)
NO	Anatomy	Board game	Thailand, 2021	To analysis students participation and experiences around the gamification process	Creating a fun-filled and interesting learning environment, improving students performances significantly.	(50)
Kahoot	Immunology	Online game	Sri lanka, 2022	To explore the medical students' perception using gamification teaching	Increasing the focus, understanding of the subject, helping retain knowledge, motivating students to learn and keeping them active throughout.	(51)
Stud2yBuddy	Dermatology	Card-based board game	UK, 2019	To develop an effective interactive resource, improve students confidence, encourage peer feedback and self-assessment of student in finals revision.	Increasing students confidence in revising dermatology.	(52)
Table-top	Emergency	Offline game	Spain, 2022	To evaluate the learning process of students and measure their knowledge improvement.	Improving medical studies and promoting knowledge retention	(53)
NO	Mental illness	Online game	Iran, 2019	To evaluate the implementation of a mental gamification and its efficacy on students.	Shaping the students' satisfaction and promoting teaching effect.	(54)
Escape boxes	Emergency	Offline game	America, 2022	To determine the effectiveness of the game in emergency medicine	Promoting teamwork and communication, improving didactics ratings	(55)
Spaced education (SE)	Anatomy, histology, cardiology and endocrinology	Online game	America, 2012	To investigate the effectiveness of the game on improving students knowledge	Having the potential to assess students knowledge and acting as an effective and well-accepted teaching means	(56)
Kaizen-IM software	Graduate education	Online game	America, 2013	To assess acceptance of the game and to determine retention of information presented to participants	Teaching critical medical concepts	(57)
East EMWars	Emergency	Longitudinal game	America, 2022	To investigate the impact of the game in emergency medicine residency training	Improving residents motivation, engagement, and challenge level	(58)
NO	Geriatric medicine	Electronic questionnaire	Switzerland, 2021	To evaluate the feasibility of the game on polypharmacy	Enhancing the attitudes and understanding of students	(59)
Escape room	Clinical practice	Offline game	America, 2019	To measure the effectiveness of the game intervention	Improving players clinical capacity and providing them with the opportunity to document event reports in real time	(60)

As an important supplement to medical curriculums, gamification has been widely used by a variety of specialties in undergraduate courses, postgraduate teaching, and resident medical education. In most cases, gamification changes traditional tedious formats of teaching activity in the undergraduate stage. Microbiology is one of the core courses in undergraduate medical education (UME) that contribute to understanding and diagnosing various clinical diseases. The application of gamification in microbiology course is a breakthrough and an innovative attempt. By exploiting a supplemental question bank that integrates game elements and clinical pearls, Walker et al. explored the effectiveness of gamification in microbiology teaching (47). In brief, the question bank resembles “tutor mode” to deliver and provide content, and students need to answer related questions to obtain the score. The composite measures include questions answered, their accuracy, and the time of response of players. This gamification design leads to higher class exam scores and increases students' basic knowledge (47). In addition, the utilization of checkerboard games also fosters the learning process and students' cognition in microbiology courses (48). A well-designed gamification process is likewise crucial for creating a fun-filled atmosphere and platform to further enhance the learning experiences and educational performance of medical students in the anatomical curriculum (49, 50).

As for other subjects in the undergraduate stage, such as immunology and dermatology, gamification of learning shows meaningful results in terms of maintaining students' attention and keeping them in an active state throughout the course (51, 52). Kahoot represents an emerging game-based learning platform that can provide timely feedback for players and is extensively applied for formative assessment during remote teaching of immunology. In the game, participants compete with each other for the correct answer and response time, and based on these performances, the overall winners will be displayed on scoreboards (51). Stud2yBuddy is a card-based board game with four categories that incorporates peer feedback and self-assessment and has the potential to offer the opportunity for improving students' content understanding (52). Moreover, gamification of learning can also shape students' satisfaction and increase their study motivation in teaching activities (53–55).

As an innovative approach for enhancing teaching effects, gamification also plays a significant role in postgraduate medical education. An online game, termed “spaced-education,” covers both preclinical and clinical domains and functions by incorporating adaptive game mechanics into an evidence-based format (56). In the game, students repeated answers to given questions according to their response accuracy to optimize long-term retention of learning. It becomes a valid and reliable tool for assessing students' knowledge and represents a well-accepted way of teaching core content (56). Furthermore, gamification together with spaced repetition studies is gradually becoming an optimal complementary method to encourage medical education and manifest obvious benefits in promoting the retention of clinical understanding (61). Likewise, the application of gamification gains well-acceptance and yields positive implications among residents (57–60).

In the context of the COVID-19 pandemic, O'Connell et al. developed a novel virtual game for obstetric and gynecology teaching. The game contains a warm-up activity and several rounds of rapid-fire questions and cases, and the main purpose of each round is to test players' knowledge about obstetric and gynecological care. This gamification attempt boosts resident education and engagement due to its entertaining, effective, and educational concepts (62). In addition, some optimized suggestions and solutions have been proposed for better designing gamification in the field of medical education during the COVID-19 era. First, teaching how to play the game by dividing lessons into smaller ones is a feasible method to develop short tests for tracking students' progress. Second, educators should take into account how students work together, their true capacities, and their preferences. Third, test design is an important element that can build students' knowledge and should be carefully treated. Furthermore, the use of different game platforms can motivate better interaction of players to improve their competition sense and sociability. Finally, future investigation also should focus on how and under what conditions gamification can maximally exert its promoting education functions effectively (63, 64).

In short, the application of gamification in medical teaching activity is promising and attractive because of its potential to provide more intuitive user experiences, solve the difficulty of remote teaching, and improve learners' engagement and motivation (65–67). Nevertheless, it is worth noting that the theoretical framework specific to medical education is still lacking, and optimized strategies as mentioned earlier deserve to be considered and further evaluated.

## 5. Discussion

Game-based learning activities can create a fascinating learning environment for students to improve their study outcomes. The benefits of GBL on learners include enhancing their collaborative awareness, providing them with opportunities for active learning to better solve clinical problems, and improving their clinical reasoning and decision-making skills. Furthermore, GBL can enable educators to explore novel and feasible teaching strategies, which contribute to the reformation of current didactical activities. Therefore, the application of serious games and gamification in medical education is meaningful.

Despite the obvious benefits, disappointing results and weaknesses remain. For example, GBL cannot yield significant outcomes in short-term gains and long-term knowledge retention sometimes (68). Therefore, how to better use recreational factors to promote teaching activity is a problem we need to solve. Moreover, there is no standardized evaluation system to measure the specific impact of GBL on students' performance and pedagogical outcomes. Many of the published works in medical education mainly converged on the engagement level and satisfaction of participants as well as the changes in knowledge score from pre-test to post-test, whereas largely ignored advanced learning objectives such as long-term knowledge gain. In the Kirkpatrick Model, the evaluation system is categorized into four levels: how participants are reacting to the program, what they can learn from the program, whether and how the program changes their practice behavior, and the profound influence of the program on participants (69, 70). In this regard, further studies should focus on assessing participants' practice behavior and the profound influence of GBL on students, which resembles the third and fourth levels of the Kirkpatrick Model. Also, most studies regarding GBL in medical education were conducted based on small sample size, and in the future, the results should be validated by further multicentric studies. As for the cost of game development, researchers should synthetically consider to best achieve the professional result of which students are used to. Finally, games are typically not considered mainstream material in medical teaching, and it is important to improve their popularizing rate and explore their potential explanatory mechanisms.

## 6. Conclusion

At present, medical education is rapidly evolving. Meanwhile, game-based learning has been gradually used for education, and several innovations have emerged. The emergence of serious games and gamification provides alternative approaches for educators to improve the medical teaching process. In most conditions, these teaching formats are well-received by learners and can create an immersive experience for students, considered effective, engaging, easy to understand, interesting, and educational in comparison with traditional teaching activities. Multiple teaching modalities of GBL also contribute to its further application, such as card, board, and even digital games using modern technology. As such, GBL has been recognized as a potential tool for enhancing medical education.

In summary, as a novel and promising teaching method, GBL has gradually become a popular addition to medical education curricula. It functions by providing the possibility of combining learning activities such as feedback, testing, and spaced repetition with active participation and autonomy as well as positive experiences for students. Designing a more effective GBL has the potential to bring an immersive experience for medical students and improve their study outcomes.

## Author contributions

HQ and XZ offered the main direction and significant guidance of this manuscript. MX and YL drafted the manuscript and made the tables for the manuscript. YZ and RX critically revised the manuscript. All authors approved the final manuscript.
